# A Nurse in General Practice

**Published:** 1968-10

**Authors:** R. W. Morris


### A NURSE IN GENERAL PRACTICE

BY

R. W. MORRIS

The recent change in the conditions of service for general practice has made auxiliary staff a comparatively inexpensive luxury. We were in course of converting a new surgery premise, and decided to base this round two consulting rooms, each with an examination couch for simple examinations, and one treatment/examination room for major examinations and other procedures. This new building was designed to house a practice of two doctors and four and a half thousand patients. On moving into the new building we adopted a full appointment system and resolved to undertake ourselves as much in the way of diagnostic procedures and minor surgery as possible. To help us we recruited two receptionists and a nurse, the latter being a mature person who had experience both as a ward sister and of working in a minor treatment unit. We allowed an initial working in period, and then recorded all work undertaken by her for six months. It was our intention to assess not only her general usefulness to us, but also whether some of our work could be delegated to her and performed at a more economic rate than if it were performed by a doctor.

We found that we could classify her work into three groups. Firstly that performed entirely by her, the only precaution being taken was that one of us was always on the premises. This work included inoculations, vaccinations, dressings, and ear syringing. She also tested all urine specimens, and organised collection of midstream urines.

The second group can be described as " assisting doctor ". This included preparing patients for examination and being present during examination, the taking of smears, and minor operations such as the removal of cysts and the tapping of hydroceles. All this saved a great deal of our time as it was never necessary to explain what clothing to remove, nor did we have to get out, put away, clean or sterilize any instruments ourselves. In fact it has proved a great joy to know that any any moment all is in readiness in the treatment room to carry out any procedure, and that one can walk out leaving someone else to clear up the mess !

The third group can be described as " general usefulness ". She is responsible for keeping the treatment room properly stocked with dressings, and organising the preparation of all repeat prescriptions. It has also become common for patients, usually women, to come into the surgery specifically for a talk with the nurse. Some problems she will deal with entirely herself, such as advice on baby feeding, while others she will refer to one of us. For example some women prefer to " break the ice " of some gynæcological symptom by talking first to her. Furthermore, it is sometimes helpful for her to sit down with a patient after the latter has seen the doctor and explain in more detail what he meant—this commonly happens with a patient starting on the " Pill ".

WORK LIST FOR THE SIX MONTHS

| Vaccs. | Injections | Dressings | Ear Syringing | Assisting Dr. Exam, smears, etc. | Total |
|---|---|---|---|---|---|
| 138 | 572 | 200 | 38 | 321 | 1,269 |

Apart from these specific duties she also acts as a third member of staff, and takes her turn to be receptionist at our one-hour Saturday morning surgery. At holiday time and during illness she does extra hours as a receptionist, our premises being open all day from 9.0 a.m. to 6.30 p.m., and the telephone is manned by staff for the booking of appointments, etc.

Without a doubt there are snags for the unwary. Before commencing work she insured herself with the General Nursing Council against legal liability. We also have an attached midwife and health visitor, and if great tact were not used all round there could well be overlapping of responsibilities, and a " nurses' war " could soon develop. It is also necessary not to overload her. We have found that it is best if she also has an appointment list in our main book, which ties in, where necessary, with both the doctors' lists. She must be lightly booked so that she is able to accommodate an examination on the spur of the moment. Our receptionist has found this complication easy to organise.

Finally there is the financial side. Her hours are from 8.45 a.m. to noon five days a week, which covers our 9-11.0 a.m. morning surgery with comfortable lee-way either side, plus two hours on every third Saturday morning. For this she is paid a monthly salary of £35. Also to be taken into account is the employers' share of the National Insurance and S.E.T. stamp. The total cost for the six month period was £243/18/2. On the credit side there is the partial reimbursement by the Executive Council, which amounted to £149/3/10, making the net cost for the period £93/14/4. In fact this works out at under £100 per partner per year, a sum which we find very reasonable. Furthermore, it should be remembered that she performed all the inoculations, so she was responsible for earning for the practice the sum of £246/15/0 in six months. We have found, therefore, that to employ a nurse is a profitable undertaking, both professionally and financially.

